# A Split‐Face Micro‐Needling Study to Evaluate the Efficacy and Consumer Perception of a Novel Moisturization Agent

**DOI:** 10.1111/jocd.70109

**Published:** 2025-03-18

**Authors:** Tahreem Nawaz, Jinseob Shin, Michelle Shieh, Jane Y. Yoo

**Affiliations:** ^1^ Clinical Research Center of New York New York New York USA; ^2^ AMOREPACIFIC US, Inc. New York New York USA; ^3^ Icahn School of Medicine at Mount Sinai New York New York USA

**Keywords:** ceramides, skin barrier, Transepidermal water loss, wound healing

## Abstract

**Background:**

Wound healing is essential for restoring skin integrity following damage. The skin barrier plays a critical role in protecting against infection, preventing moisture loss, and supporting regeneration. Ceramides, integral components of the lipid matrix, are known to improve skin hydration, reduce inflammation, and accelerate wound healing. However, research on ceramide‐based formulations in post‐procedural settings remains limited.

**Aims:**

This study aims to evaluate the efficacy of Aestura ATOBARRIER 365 Cream, containing a Lipid Complex with ceramides, cholesterol, and fatty acids, in promoting skin barrier recovery and improving outcomes after barrier disruption.

**Methods:**

A randomized, double‐blind, split‐face trial was conducted with 30 participants aged 22–60 years. Following microneedling, the active formulation was applied to one side of the face and the vehicle formulation to the other, twice daily for 4 weeks. Transepidermal Water Loss (TEWL), erythema, and roughness were measured at baseline, post‐application, and at weeks 2 and 4. Participant surveys assessed moisturization, erythema, and overall skin improvement.

**Results:**

Both formulations demonstrated significant reductions in TEWL (14%–16%) and erythema (~1.7%) by week 4 compared to post‐microneedling. High participant satisfaction was also observed, with 93% reporting improved adequate skin hydration and 90% reporting reduced erythema with the active formulation.

**Conclusions:**

Aestura ATOBARRIER 365 Cream with ceramides demonstrated efficacy in improving skin barrier recovery, reducing TEWL, and enhancing skin texture following microneedling. The significant reductions in TEWL, erythema, and roughness highlight its ability to restore the skin's barrier, calm irritation, and refine texture. These findings confirm its role in supporting skin recovery and resilience after dermatological treatments, making it a valuable addition to post‐procedure care. While these results support its effectiveness in post‐procedural recovery, further research is needed to determine its applicability for other conditions involving compromised skin barriers, such as eczema or rosacea. Additional studies are also warranted to assess long‐term efficacy and its potential role in optimizing skin barrier restoration across diverse patient populations.

## 1 Introduction

Wound healing is a complex and vital process that is essential for restoring skin integrity following injury. The skin's barrier function plays a crucial role in protecting against infection, minimizing moisture loss, and supporting tissue regeneration. When the skin barrier is compromised during injuries or certain cosmetic and medical procedures, it becomes more susceptible to external irritants and dehydration, which can delay healing and lead to complications [1, 2]. Accelerating the healing process while restoring the skin's barrier is a key focus in dermatological care, making moisturizers and wound healing products essential for post‐injury care.

Ceramides are a class of lipids that are integral to the skin's barrier function, helping to retain adequately hydrated skin and prevent transepidermal water loss (TEWL) [3, 4, 5]. Studies have demonstrated that ceramide‐containing formulations improve skin barrier function, reduce inflammation, and promote more rapid epithelial recovery in wounded skin [6, 7]. Ceramides are clinically relevant in conditions like eczema, where a compromised skin barrier leads to dryness and inflammation. Studies show that ceramide‐based treatments help restore barrier function, reduce inflammation, and promote faster wound healing by enhancing skin hydration [8, 9, 10, 11]. This makes ceramides a critical component in managing eczema and other conditions associated with impaired barrier function. Ceramide deficiency is also linked to delayed wound healing and increased sensitivity to environmental irritants, further highlighting the importance of ceramides in skin recovery [5].

Despite their well‐documented benefits in dermatological conditions, there remains limited research on the efficacy of ceramide‐based formulations in post‐procedural recovery. Most studies have focused on general wound care, leaving a gap in understanding their role following cosmetic procedures that disrupt the skin barrier. ATOBARRIER 365 Cream incorporates UHD DermaON, a structured ceramide complex designed for optimized penetration and barrier reinforcement, which has been shown to improve lipid organization and enhance skin retention of active ingredients [12]. Given its unique formulation, this study aims to evaluate its effects specifically in the context of microneedling, contributing to the growing body of research on ceramides in post‐procedural settings.

This study aims to investigate the efficacy of Aestura ATOBARRIER 365 Cream, which contains an advanced Lipid Complex with ceramides, cholesterol, and fatty acids, in supporting wound healing and enhancing skin barrier recovery. The cream will be applied post‐microneedling, a procedure that temporarily disrupts the skin barrier. By conducting a split‐face trial comparing the cream with and without the active Lipid Complex, this study will provide insights into the potential benefits of ceramide‐enriched formulations for wound healing.

## 2 Methods and Materials

### 2.1 Study Design

This split‐face, randomized, double‐blind clinical trial was designed to evaluate the efficacy of Aestura ATOBARRIER 365 Cream with and without the active Lipid Complex in enhancing skin barrier recovery and improving post‐microneedling wound healing. Randomization for the left or right side of the face for each participant was performed using a computer‐generated randomization table. The study was double‐blinded, with participants unaware of the product allocation, while the evaluator did not have access to the randomization table until the end of the study. The study was conducted in accordance with Good Clinical Practice (GCP) guidelines and was approved by the Advarra Institutional Review Board (IRB). Written informed consent was obtained from all participants prior to study enrollment.

### 2.2 Participants

A total of 31 participants were initially enrolled, and 30 participants completed the study. One participant withdrew due to personal reasons, and five participants were discontinued from image analysis because their beards interfered with color assessments. However, these participants continued with other study assessments. All statistical analyses were conducted on 30 participants (*N* = 30). Subjects were aged 22 to 60 years, with Fitzpatrick skin types I–VI. Participants were excluded if they had pre‐existing dermatological conditions that could affect the study results or known allergies to any ingredients in the cream. Specific exclusion criteria included recent use of cosmetic procedures, laser treatments, or chemical exfoliants. The study participants were members of the general public who enrolled in response to a participant recruitment call issued by Eurofins CRL (ECRL), a clinical research organization. These individuals were not hospitalized patients but volunteers from the community who met the study's eligibility criteria.

### 2.3 Intervention

Each participant underwent microneedling on the full face using a standardized microneedling device with a 1.5 mm needle length. Immediately following the procedure, participants were randomly assigned to receive Aestura ATOBARRIER 365 Cream with the active Lipid Complex (Product A) on one side of the face and Aestura ATOBARRIER 365 Cream without the active Lipid Complex (Product B) on the other side. The active Lipid Complex in Product A is a capsulized ceramide marketed as DermaON and referred to as UHD DermaON in research reports [12, 13, 14].

The creams were applied in equal amounts, and participants were instructed to use the respective creams twice daily for 4 weeks post‐procedure. Compliance was monitored through daily diaries. Participants were monitored throughout the study for any adverse effects. Any reactions, such as irritation or allergic response, were recorded and reviewed by the study's dermatologist.

### 2.4 Outcome Measures

The primary outcome measures included TEWL, skin erythema, skin roughness, and overall skin barrier recovery. All outcome measures were collected by expert clinicians who visually assessed and graded skin conditions at baseline, immediately post‐application (within 15 min of cream application), and at weeks 2 and 4. TEWL was measured using a VapoMeter, a non‐invasive device that assesses water loss from the skin. Expert clinicians graded erythema and roughness visually, using a validated 4‐point scale. Participants were asked to complete a self‐assessment questionnaire at baseline, week 2, and week 4. Questions included their perceptions of skin hydration/moisture, erythema, flakiness, and overall skin improvement.

### 2.5 VISIA‐CR Image Analysis

VISIA‐CR imaging technology was used to assess skin erythema, lightness, and color homogeneity following microneedling and product application. Cross‐polarized images of participants' faces were captured at baseline, post‐microneedling, post‐product application, and at weeks 2 and 4. These images were analyzed using the CIE Lab color space to quantify erythema (a*) and lightness (L*), where L* specifically represents the light‐to‐dark spectrum in objective skin color analysis. In addition, Individual Typological Angle (ITA°) and Individual Whitening Angle (IWA°Newtone) were calculated to evaluate pigmentation and whitening effects.

### 2.6 Statistical Analysis

Data were analyzed using paired t‐tests and analysis of variance (ANOVA) with Dunnett's test to compare the change from baseline, post‐microneedling, and the difference between Product A and Product B at each time point. Statistical significance was set at *p* < 0.05. All statistical analyses were performed using professional statistical software.

## 3 Results

### 3.1 Transepidermal Water Loss (TEWL)

TEWL demonstrated significant improvements in skin barrier function following the application of both formulations of Aestura ATOBARRIER 365 Cream. For the group using Product A, TEWL measurements showed a reduction of 14.2% at week 2 (*p* < 0.0001, 95% CI [−18.6, −9.81]) and 15.0% at week 4 (*p* < 0.0001, 95% CI [−19.3, −10.6]) compared to post‐microneedling values. Similarly, Product B also resulted in decreased TEWL, with improvements of 15.3% at week 2 (*p* < 0.0001, 95% CI [−22.0, −8.49]) and 16.3% at week 4 (*p* < 0.0001, 95% CI [−23.0, −9.51]). The differences between the active and vehicle formulations were not statistically significant at the 95% confidence level. The reduction in TEWL demonstrates the formula's effective enhancement of the skin barrier's ability to retain adequately hydrated skin. This improvement is notable given the increased skin permeability typically associated with microneedling, highlighting the formula's role in accelerating skin recovery and restoring barrier function after a dermatologist‐administered treatment.

### 3.2 Erythema

**FIGURE 1:**
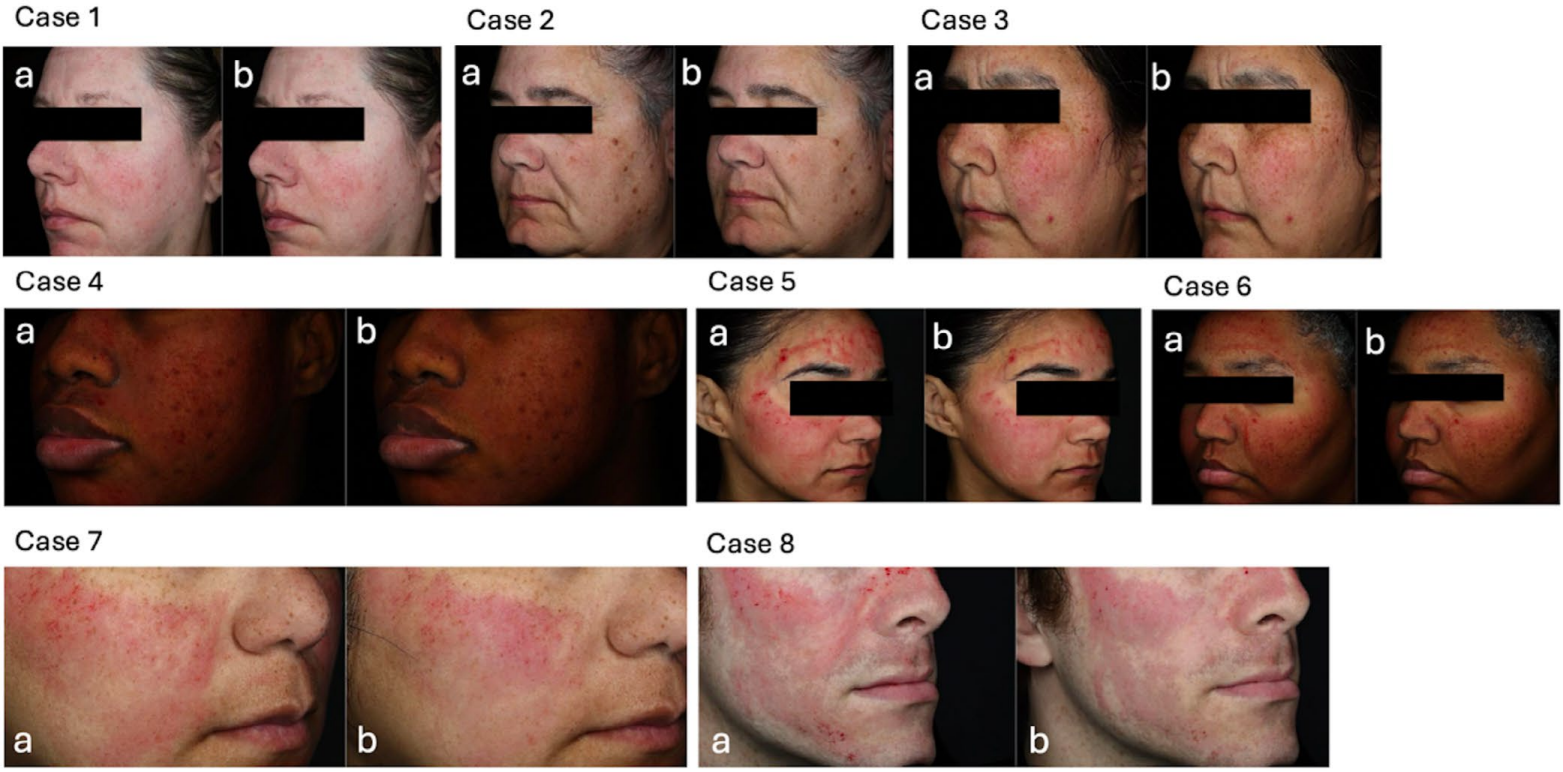
Case examples showing the effects of Product A (active formulation) immediately post‐microneedling and after application. Each case [1 through 8] presents side‐by‐side images: (a) post‐microneedling and (b) immediately after applying the active formulation.

In the group using Product A, erythema decreased by 1.8% at week 2 (*p* < 0.0001, 95% CI [−2.55, −1.11]) and 1.7% at week 4 (*p* < 0.0001, 95% CI [−2.45, −1.01]), indicating a consistent reduction in erythema over time. The gradual reduction in erythema, as assessed by clinical experts, underscores the formula's effectiveness in reducing post‐procedure erythema. This improvement suggests that the formula aids in calming and soothing the skin, making it an effective option for managing erythema and discomfort following microneedling. Product B demonstrated similar results, with reductions in erythema of 1.7% at both week 2 (*p* < 0.0001, 95% CI [−2.38, −1.08]) and week 4 (*p* < 0.0001, 95% CI [−2.32, −1.02]). VISIA‐CR image analysis further supported these results, showing a quantitative reduction in erythema (a) by 1.97% immediately after application (*p* < 0.0001, 95% CI [−2.66, −1.28]) and 8.61% at week 2 compared to post‐microneedling (*p* < 0.0001, 95% CI [−10.6, −6.62]), with a reduction of 8.56% by week 4 (*p* < 0.0001, 95% CI [−10.5, −6.64]). Figure 1 visually demonstrates the immediate reduction in erythema post‐application of Product A. Although both formulations showed statistically significant improvements compared to post‐microneedling, no statistically significant differences were observed between the active and vehicle formulations.

### 3.3 Roughness

For participants using Product A, roughness decreased by 0.6% at week 2 (*p* = 0.0460, 95% CI [−1.13, −0.007]) and 0.7% at week 4 (*p* = 0.0126, 95% CI [−1.23, −0.11]) compared to post‐microneedling measurements. The observed decrease in skin roughness indicates that the formula contributes significantly to smoothing and refining skin texture. This improvement is especially significant in the context of post‐microneedling skin, where the texture may initially be uneven. The formula's efficacy in improving skin smoothness suggests it supports the skin's healing process by enhancing overall texture and appearance. Product B showed a slightly lesser reduction, with decreases of 0.5% at week 2 (*p* = 0.0430, 95% CI [−1.05, −0.01]) and 0.6% at week 4 (*p* = 0.0275, 95% CI [−1.09, −0.05]) compared to the post‐microneedling measurements. Overall, both formulations significantly decreased skin roughness, with the active formulation providing marginally better results, particularly at week 4.

### 3.4 Color Evolution

#### 3.4.1 Color Evolution Post‐Microneedling

**FIGURE 2:**
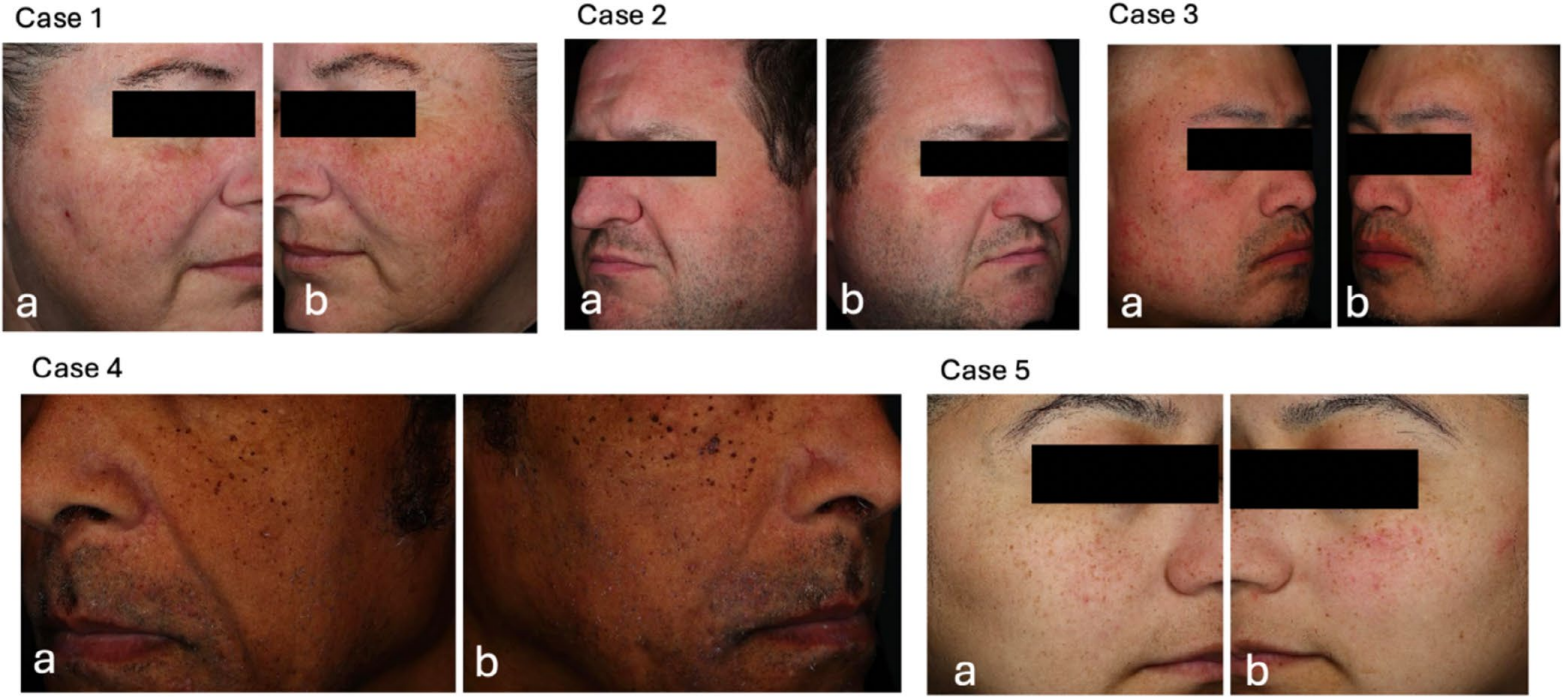
Case examples showing the effects of Aestura ATOBARRIER 365 Cream after 4 weeks of application. Each case [1 through 5] presents side‐by‐side images: (a) 4 weeks after using Product A (active formulation) and (b) 4 weeks after using Product B (vehicle formulation).

The VISIA‐CR analysis revealed the effects of the active and vehicle formulations on skin color parameters after microneedling. For Product A, skin lightness (L*) significantly increased by 1.08% immediately after application compared to post‐microneedling (*p* < 0.0001, 95% CI [0.580, 1.58]) [15]. Erythema (a*) showed a significant reduction of 1.97% immediately post‐application (*p* < 0.0001, 95% CI [−2.66, −1.28]). By week 2, the improvements were more substantial, with a 7.00% increase in skin lightness (*p* < 0.0001, 95% CI [5.02, 8.97]) and a 8.61% reduction in erythema (*p* < 0.0001, 95% CI [−10.6, −6.62]) compared to post‐microneedling. At week 4, the improvements continued, with a 5.01% increase in skin lightness and an 8.56% reduction in erythema (*p* < 0.0001, 95% CI [3.23, 6.78] for lightness and *p* < 0.0001, 95% CI [−10.5, −6.64] for erythema). These results show a steady improvement in skin tone and color uniformity after treatment with the active formulation (Figure 2).

For Product B, a similar trend was observed. Skin lightness (L*) significantly increased by 1.31% immediately after application (*p* < 0.0001, 95% CI [0.664, 1.96]), while erythema (a*) significantly decreased by 2.23% (*p* < 0.0001, 95% CI [−3.00, −1.47]). By week 2, lightness had improved by 6.28% (*p* < 0.0001, 95% CI [4.51, 8.06]) and erythema decreased by 8.65% (*p* < 0.0001, 95% CI [−10.6, −6.71]). At week 4, these improvements remained consistent, with a 5.16% increase in lightness (*p* < 0.0001, 95% CI [3.40, 6.93]) and an 8.64% reduction in erythema (*p* < 0.0001, 95% CI [−10.6, −6.63]), compared to post‐microneedling.

Both formulations demonstrated significant improvements in skin lightness and erythema immediately after application, with these effects becoming more pronounced over time.

#### 3.4.2 Color Evolution From Baseline

**FIGURE 3:**
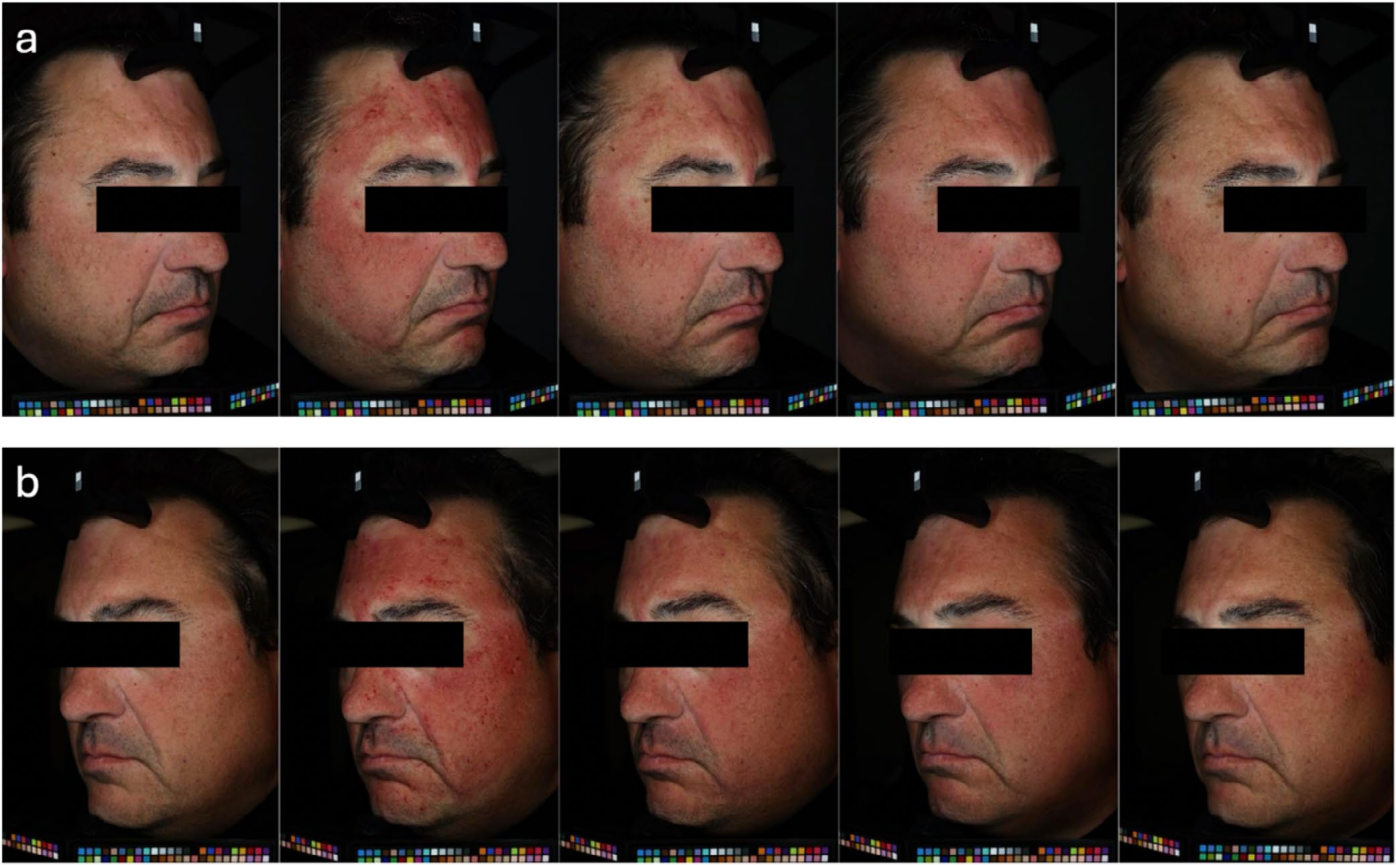
Longitudinal case example showing the effects of Aestura ATOBARRIER 365 Cream on (a) one side of the face treated with Product A (active formulation) and (b) other side treated with Product B (vehicle formulation). Five time points are presented: Baseline, post‐microneedling, post‐product application, week 2, and week 4.

Color changes from baseline were also assessed, with Figure 3 depicting a longitudinal case example. For Product A, skin lightness (L*) significantly decreased by 1.64% at week 2 compared to baseline (*p* = 0.0002, 95% CI [−2.42, −0.853]), while erythema (a*) increased by 0.64% (*p* = 0.033, 95% CI [0.0557, 1.23]). By week 4, a 3.62% reduction in skin lightness was observed (*p* < 0.0001, 95% CI [−4.72, −2.53]), while erythema increased by 0.69%, though the change in erythema was statistically non‐significant (*p* = 0.0650, 95% CI [−0.0467, 1.43]).

For Product B, skin lightness significantly decreased by 1.78% at week 2 (*p* = 0.0002, 95% CI [−2.63, −0.927]), and erythema increased by 0.37% compared to baseline, with no statistical significance (*p* = 0.283, 95% CI [−0.328, 1.07]). At week 4, lightness significantly decreased by 2.90% (*p* < 0.0001, 95% CI [−3.69, −2.11]), while erythema increased by 0.38% from baseline, without statistical significance (*p* = 0.313, 95% CI [−0.386, 1.15]).

However, both products demonstrated significant improvements in pigmentation and skin homogeneity over time. For Product A, there was an 8.39% improvement in ITA° (*p* < 0.0001, 95% CI [−11.0, −5.75]), and a 1.89% improvement in IWA°Newtone by week 4 (*p* < 0.0001, 95% CI [−2.65, −1.13]), while Product B showed a 6.71% improvement in ITA° (*p* < 0.0001, 95% CI [−8.65, −4.76]), and a 1.38% improvement in IWA°Newtone (*p* = 0.0006, 95% CI [−2.10, −0.655]). These findings indicate that, although there were some fluctuations in skin lightness and erythema from baseline, both formulations produced significant improvements in skin pigmentation and homogeneity by week 4.

### 3.5 Participant‐Reported Outcomes

Participant‐reported outcomes were collected via self‐assessment questionnaires administered at baseline, week 2, and week 4. These subjective evaluations showed high levels of satisfaction among participants. At week 4, 93% of participants using Product A reported feeling that their skin was well‐moisturized, compared to 96.7% of those using Product B. 90% of participants using Product A reported a noticeable reduction in post‐microneedling irritation and erythema, whereas 90% and 93% of participants using Product B reported similar improvements in irritation and erythema, respectively. Overall satisfaction with both products was high, with 93% of participants expressing satisfaction with Product A, compared to 90% for Product B.

Participant‐reported outcomes demonstrated high satisfaction with both formulations, particularly regarding moisturization, irritation reduction, and overall skin improvement. While these subjective reports align with the improvements seen in TEWL and VISIA‐CR imaging, it is important to consider the potential influence of placebo effects. Given that participants were aware of using two different formulations, their perceived differences in hydration and erythema reduction may have been partially influenced by expectation rather than actual physiological changes. However, the significant reductions in TEWL and erythema observed through objective measurements for both formulations provide strong validation of the cream's effectiveness beyond self‐reported perception.

### 3.6 Safety

Both formulations were well‐tolerated throughout the study, with no significant adverse events reported. None of the participants experienced severe irritation, allergic reactions, or other complications.

## 4 Discussion

### 4.1 Main Results

This study aimed to assess the efficacy of Aestura ATOBARRIER 365 Cream, including its Lipid Complex containing DermaON (active ingredient), in promoting skin barrier recovery. Results indicate that both formulations (Product A and Product B) improved TEWL, reduced erythema, and improved skin texture post‐microneedling. VISIA‐CR analysis confirmed significant reductions in erythema, lightness, and improved skin homogeneity, supporting the efficacy of both formulations in enhancing skin recovery post‐procedure. However, Product B showed slightly superior results in TEWL reduction, although there was no statistically significant difference between the two formulations. Product A showed marginally better results in skin texture improvements.

Participant satisfaction was high across the board, with 93% reporting significant improvements in adequately hydrated skin and 90% reporting significant improvements in erythema. Both clinical grading and participant self‐reports aligned, showing consistency in product efficacy.

### 4.2 Skin Barrier Recovery and Wound Healing

The DermaON, which is a key component of the Lipid Complex, played a significant role in accelerating skin barrier recovery. Ceramides are known to improve the skin's barrier function by reinforcing lipid layers, helping retain adequately hydrated skin, and promoting faster epithelial regeneration [13, 14]. Previous studies, including both ex vivo and in vitro trials, demonstrated the efficacy of DermaON in strengthening the barrier by enhancing the formation of claudin‐4 proteins, which are crucial for tight junctions and cell adhesion [13, 14]. This explains the significant reductions in TEWL and enhanced moisture retention seen in this study.

The study results align with previous research on ceramides, showing that lipid complexes containing ceramides not only improve hydration but also promote faster wound healing by creating a more effective barrier to prevent TEWL [13]. The in vitro study demonstrated a significant increase in claudin‐4 (CLDN4) gene expression, further indicating the efficacy of DermaON in enhancing barrier recovery [13, 14]. The ex vivo penetration studies confirmed that DermaON particles penetrated and remained within the skin's stratum corneum for up to 18 hours, providing sustained barrier support [14].

### 4.3 Consistency

These results align with existing literature on ceramide‐based formulations, particularly in post‐procedural skincare. Studies have shown that ceramides help restore the skin barrier following damage caused by procedures like microneedling or laser treatments, making them a key ingredient in promoting recovery [16]. In this trial, the use of DermaON significantly enhanced the skin's ability to reduce water loss and protect against irritants, like findings from studies on other ceramide‐based formulations used for conditions such as atopic dermatitis [9, 17].

### 4.4 Clinical Implications

Given these findings, the Aestura ATOBARRIER 365 Cream can be considered an effective post‐microneedling recovery product. Its ability to accelerate skin barrier recovery, reduce erythema, and improve skin texture suggests that it may be beneficial for patients undergoing the procedure. Moreover, the high participant satisfaction, including improved moisturization and reduced erythema, supports the efficacy of this cream in post‐microneedling recovery. While the findings suggest potential benefits for other dermatological treatments involving barrier recovery, additional studies are needed to confirm its applicability beyond post‐procedural care. Similarly, although the active formulation has demonstrated moisturization and wound‐healing capabilities, further research is required to determine its effectiveness in managing skin conditions such as atopic dermatitis and psoriasis.

While statistical significance is a key factor in evaluating treatment efficacy, it is equally important to assess whether the observed changes provide meaningful benefits in clinical practice. The reduction in TEWL (14.2% at week 2 and 15.0% at week 4 for the active formulation) suggests enhanced skin barrier function, which may contribute to reduced moisture loss and improved hydration, which are critical factors in post‐microneedling recovery. However, both formulations demonstrated statistically significant TEWL reductions of 14%–16%, making it difficult to determine whether the active formulation provided additional benefits beyond the hydrating effects of the vehicle.

Research shows that reducing TEWL improves skin hydration, resilience, and barrier function, helping protect against irritants and support cell turnover. A placebo‐controlled study on krill oil supplementation further validated this, demonstrating that TEWL reduction correlated with improved skin hydration and elasticity, with results significantly differing from the placebo group. This reinforces that TEWL reduction is not just a statistical measure but an indicator of real physiological benefits [18].

Similarly, the reduction in erythema (1.8% at week 2 and 1.7% at week 4) may appear small in absolute terms but is supported by VISIA‐CR imaging, which detected an approximately 9% reduction in erythema‐related color parameters over the study period. In clinical practice, even minor reductions in erythema can improve patient comfort, particularly for individuals prone to post‐inflammatory erythema or heightened skin sensitivity after microneedling. While no significant differences were observed between formulations, both may have contributed to erythema reduction through improved skin barrier function. Studies have shown that ceramide‐based formulations help improve erythema in conditions involving a compromised skin barrier, with significant reductions observed in patients with mild‐to‐moderate atopic dermatitis and other pruritic dermatoses [9, 17, 19]. While ceramides have demonstrated anti‐inflammatory benefits, our study's four‐week duration may not have been sufficient to detect significant differences between formulations.

Furthermore, participant‐reported outcomes reinforce the clinical relevance of these findings. Despite the modest numerical differences in TEWL and erythema, 90%–95% of participants noted improved skin hydration and reduced irritation, suggesting that these changes were noticeable and beneficial in a real‐world context. Similarly, while improvements in skin roughness and color evolution were modest, smoother skin texture and a more even tone can still enhance post‐procedural recovery. For example, 97% and 93% of participants reported that their skin felt softer and healthier, respectively, by Week 4 with the active formulation, suggesting that these subtle changes contributed to a positive patient experience and perceived benefit of treatment. These findings underscore the importance of even minor textural and color improvements in post‐microneedling care. However, further research is needed to establish definitive clinical thresholds for TEWL and erythema reduction and skin texture changes in post‐procedural skincare.

### 4.5 Limitations and Future Studies

This study had several limitations, including a relatively small sample size (*N* = 30) and a follow‐up period of only four weeks. Future studies should involve larger sample sizes and longer follow‐up periods to assess the long‐term efficacy of ceramide‐based formulations like Aestura ATOBARRIER 365 Cream. Furthermore, evaluating the cream's efficacy in treating other skin conditions could provide more insight into its clinical applications.

Another limitation of this study is the absence of a traditional control group, such as an untreated area or a well‐established post‐microneedling moisturizer for comparison. Instead, the study compared two variations of the same product, both of which contained hydrating and barrier‐repairing properties. This study design may have minimized measurable differences between formulations, making it more difficult to isolate the specific effects and perhaps additional improvements of the active Lipid Complex. Future research should include a more traditional control vehicle, devoid of moisturizing properties, to better contextualize the efficacy of Aestura ATOBARRIER 365 Cream within the broader landscape of post‐procedural skincare.

Additionally, the relatively small sample size and short follow‐up period may have limited the ability to detect statistically significant differences. A longer study duration might have allowed for a more pronounced distinction between formulations.

Lastly, there is the potential for placebo effects in participant‐reported outcomes. While high satisfaction rates suggest a positive experience with both formulations, subjective responses may be influenced by perception rather than actual physiological changes. To mitigate this, objective measures such as TEWL and VISIA‐CR imaging were used to validate the findings. Future studies could incorporate additional blinded assessments or alternative methodologies to further minimize the potential impact of placebo effects on participant‐reported outcomes.

## 5 Conclusions

In conclusion, Aestura ATOBARRIER 365 Cream, including the version with the active Lipid Complex containing DermaON (Product A), demonstrated significant efficacy in enhancing skin barrier recovery, improving adequate skin hydration, and reducing TEWL. While both formulations showed statistically significant improvements, the minimal differences between them suggest that the vehicle's hydrating properties may have contributed to the observed effects. This product presents an effective option for post‐microneedling skincare, with potential applications in other dermatology treatments to improve skin barrier function.

However, further research is warranted to assess the long‐term outcomes of ceramide‐based formulations, as this study was limited to a four‐week follow‐up period. Additionally, evaluating potential limitations in the formulation, such as its performance under different environmental conditions or in combination with other skincare regimens, could provide valuable insights. Future studies should also investigate whether similar improvements in skin barrier function and wound healing are observed in individuals with various dermatological conditions, such as eczema or rosacea, and across diverse skin types. Expanding this research will help refine the application of ceramide‐enriched skincare for broader clinical use.

## Author Contributions

All authors contributed to the conception and design of the study, ensuring alignment with the study's objectives and scientific rigor. They participated in various stages of execution, including protocol development, subject recruitment, data collection, and analysis. Additionally, all authors reviewed and revised the manuscript, providing critical insights to ensure the accuracy and clarity of the findings.

## Ethics Statement

The authors confirm that the ethical policies of the journal, as noted on the journal's author guidelines page, have been adhered to and the appropriate ethical review committee approval has been received. The study protocol was reviewed and approved by the Advarra Institutional Review Board (IRB), 6100 Merriweather Dr., Suite 600, Columbia, MD 21044 (Approval in 4/2024). The authors confirm that all participants signed a photography release form, allowing the capture and use of their images for the purposes of this clinical study.

## Consent

Written informed consent was obtained from all subjects in accordance with Title 21 of the Code of Federal Regulations (CFR), Parts 50 and 56.

## Conflicts of Interest

Dr. Jane Y. Yoo serves as a consultant for Amorepacific US Inc. Jinseob Shin and Michelle Shieh are employees of Amorepacific US Inc.

## Data Availability

The data that support the findings of this study are available on request from the corresponding author. The data are not publicly available due to privacy or ethical restrictions.
